# Multi-level treatment outcome evaluation in adolescents with autism spectrum disorder

**DOI:** 10.1186/s13034-025-00909-1

**Published:** 2025-05-19

**Authors:** Gabriel Anton Auer, Paul Lukas Plener, Luise Poustka, Lilian Konicar

**Affiliations:** 1 Department of Child and Adolescent Psychiatry, Medical University of Vienna, Vienna, Austria; 2 Comprehensive Center for Pediatrics (CCP), Medical University of Vienna, Vienna, Austria; 3 Comprehensive Center for Clinical Neuroscience and Mental Health (C3NMH), Medical University of Vienna, Vienna, Austria; 4 Department of Child and Adolescent Psychiatry and Psychotherapy, Ulm University, Ulm, Germany; 5 Department of Child and Adolescent Psychiatry, University Hospital Heidelberg, Heidelberg, Germany

**Keywords:** Resting state EEG, Alpha brain activity, Autism spectrum disorder, Neurofeedback, Brain regulation training

## Abstract

**Background:**

Aberrant resting state electroencephalography (rsEEG) is a well-established indicator of psychopathological brain activity in clinical disorders. In Autism Spectrum Disorder (ASD), a substantial body of research reports reduced Alpha activity in the electrocortical resting state of affected individuals. However, effective interventions based on neurophysiological patterns and objective biological markers of treatment outcome remain scarce.

**Methods:**

In this randomized controlled trial, the primary objective was to examine rsEEG changes in adolescents with ASD following 24 sessions of slow cortical potential neurofeedback training (*n* = 21) compared to a treatment-as-usual control group (*n* = 20). A repeated-measures analysis of variance was used to assess group differences over time. Additionally, Pearson correlation analyses were conducted to exploratorily investigate associations between rsEEG measures and clinical psychopathology and affective well-being, as assessed via parental and self-report questionnaires at baseline and post-intervention.

**Results:**

Analyses revealed significant differences in the development of rsEEG between the intervention groups: while Alpha activity increased in the experimental neurofeedback group, it decreased in the control group, demonstrating an opposite trend. Exploratory analyses showed that Delta activity decreased in both groups, with a more pronounced decrease in the experimental group. Correlational analyses revealed significant associations between subjective-psychological and electrocortical levels: lower alpha power at baseline was related to greater severity of ASD symptoms, while both lower alpha and higher delta power were associated with greater negative affect at baseline. Increases in alpha power after NF-training were linked with enhanced positive affect, whereas reductions in delta power corresponded to decreases in negative affect.

**Conclusions:**

This study provides insights into changes in resting-state neural activity before and after clinical interventions alongside clinical-psychological assessment, overcoming single-level assessments and emphasizing the need for multi-level outcome measures for a more comprehensive treatment evaluation.

*Clinical Trial Registration*: DRKS00012339.

**Supplementary Information:**

The online version contains supplementary material available at 10.1186/s13034-025-00909-1.

## Background

Autism spectrum disorders (ASD) are a group of life-long neurodevelopmental disorders marked by difficulties in social interactions, along with restricted, repetitive behaviours and interests. Symptoms typically emerge in early childhood and can vary in severity. They often intensify during development, resulting in clinically significant impairments in daily functioning [1].

Interestingly, the prevalence of ASD has increased significantly across various countries over the past few decades [2, 3] placing a substantial health and economic burden on communities worldwide. Although factors like advanced maternal and paternal age or maternal metabolic conditions during pregnancy may have contributed to the rise in ASD prevalence [4–6], heightened awareness and improved diagnostic capabilities are likely the predominant drivers of the current trend [7].

However, despite increased awareness and progress in diagnostics, the development of adequate treatment methods lags behind. Insufficient funding for research and services as well as a lack of knowledge on evidence-based treatments have been identified as significant gaps in ASD care [8]. This is unsurprising, as evidence-based treatments for ASD are scarce. Among the multitude of treatment modalities, only a few behavioural interventions and medical therapies have been proven effective [9, 10], primarily in children up to 12 years of age as other age groups remain underrepresented in research [11]. Yet, behavioral interventions are often time- and cost-intensive [12], while psychopharmacological treatments largely target comorbid conditions [13] and carry an increased risk of side effects for children and adolescents with ASD [14]. It is therefore crucial to allocate more resources toward the exploration of treatment alternatives, while also expanding the study population beyond children.

As the symptomatology of ASD varies significantly across affected individuals [15], research on treatment methods proves somewhat difficult. Thus, focusing on underlying mechanisms, particularly neurophysiological alterations, rather than solely targeting behavioral manifestations, may offer a more effective framework for understanding and addressing the disorder [16].

Consistent evidence from structural magnetic resonance imaging (MRI) studies in ASD (summarized in Patil et al. [17]), reports a reduction in gray matter volume in regions crucial for social interactions and emotional processing, including the amygdala, the fusiform gyrus and the superior temporal sulcus. Regarding functional connectivity, research has shown reduced connectivity within the default mode network, a brain network implicated in self-reflection and theory of mind. These selected findings demonstrate that brain areas involved in social, emotional, and sensory processing - functions often impaired in ASD - are indeed frequently found to be both structurally and/or functionally altered, indicating a neurobiological foundation of the disorder’s symptomatology.

While insights from MRI studies provide valuable information, electroencephalography (EEG) has a much higher temporal resolution and measures neural activity directly, highlighting its importance as a complementary method for understanding neural processes. Furthermore, EEG does not present specific challenges inherent to MRI, such as confined spaces, loud noises, and the need to remain still, that are frequently perceived as barriers by individuals with ASD [18]. In addition to being comparably inexpensive and broadly accessible, these advantages make EEG an essential tool for examining neural processes in ASD.

Resting-state (rs) EEG, which reflects an individual’s typical neural activity during rest (i.e., when not engaged in mentally or physically demanding tasks), is one of the most established EEG-based measures, alongside event-related potentials (ERPs). However, rsEEG recordings are conducted under conditions that are oftentimes heterogeneous, thereby complicating comparability across studies. rs paradigm types (eyes-closed (EC) vs. eyes-open (EO)) vary across studies, with differences in recording durations of EC and EO segments or the inclusion of only one condition, all of which can affect the recorded data [19]. Additionally, factors such as the day of the experiment, the time of day, and the level of physical activity prior to recording - variables likely differing across studies - have been shown to impact rsEEG data [20]. Moreover, significant heterogeneity in methodological approaches and the targeting of different EEG features across studies further complicates the comparison of results. However, despite these challenging conditions, certain abnormal power band characteristics have been repeatedly observed in individuals with ASD during rs measurements.

Regarding the Delta band, increased power is frequently reported, both in absolute [21–24] and relative measures [21, 22, 25]. This increased Delta power has been observed primarily in frontal areas [23, 24], but also throughout the entire cortex [22, 25].

Similarly, concerning Theta power, higher absolute [22, 23, 26] and relative [22, 27, 28] power has recurrently been assessed, particularly in frontal, prefrontal, and midline regions [22, 23, 26, 28].

Regarding the high-frequency Beta band, some studies suggest increases in both absolute [29, 30] and relative power [30]. Furthermore, some evidence suggests increased absolute [29–31] and relative Gamma power [30, 32], specifically in frontal, central and parietal regions [31, 32].

While multiple studies have shown increased power in the low-frequency (Delta and Theta) and high-frequency (Beta and Gamma) bands, the Alpha band exhibits a contrasting pattern. Reduced absolute and relative Alpha power is a consistent and robust finding across numerous rsEEG studies in ASD [21, 22, 25, 28, 30, 33, 34, 35, 36].

Taken together, these findings suggest a U-shaped profile of rs power abnormalities in ASD, as outlined in a review by Wang et al. [37]. This profile is characterized by heightened power in both low and high frequency bands, specifically increased Delta and Theta power at the lower end, as well as Beta and Gamma power at the higher end and reduced Alpha activity, which is located in the lower-middle range of the power spectrum [37]. Despite some results contradicting the U-shaped curve hypothesis [24, 27, 38], likely due to ASD’s neurophysiological diversity and heterogeneous measurement conditions, certain power abnormalities remain consistent and robust when reviewing the current research. A recent systematic review and meta-analysis by Neo et al. [19], which included 41 studies with 1246 autistic and 1455 neurotypical individuals (with some overlap with Wang et al. [37]), partially supports the U-shaped curve hypothesis, identifying a reduction in relative rs Alpha power and an increase in both absolute and relative Gamma power in individuals with ASD. In addition, Neo et al. [19] demonstrated that the type of rs paradigm (EC vs. EO) significantly moderated effect sizes, with the EO condition producing larger effect sizes. Similarly, the duration of rs recordings moderated Alpha power effect sizes, with longer recording durations yielding more pronounced effects. These findings further suggest that the potential of rs Alpha power as a robust biomarker for ASD is particularly evident when measured under EO conditions with sufficiently long recording durations.

Although several electrocortical abnormalities have been identified in ASD, biological treatment approaches targeting neural mechanisms are still in their infancy. EEG neurofeedback (NF) is a safe, side-effect-free, biologically based treatment approach, ideally suited to address abnormal EEG activity. Research focused on children and adolescents has demonstrated its potential in conditions where such abnormalities are prevalent, including e.g. epilepsy [39, 40], anxiety [41], and trauma [42]. In the context of ASD, several recent reviews [43–46] have concluded that NF holds promise as a tool for alleviating ASD symptoms. However, as noted by Kumari & Sharma [43], the evaluation of these symptoms has predominantly relied on subjective instruments along with parental input. Given NF’s susceptibility to non-specific effects [46], incorporating objective measures alongside subjective assessments is necessary to validate its efficacy. This became particularly evident in attention deficit hyperactivity disorder (ADHD), where two recent meta-analyses found improvements in attentional performance following NF [47, 48]. However, Chung et al. [47] found no benefit over placebo when only subjective measures were used in double-blind studies, while Chiu et al. [48] observed significant improvements with objective behavioral measures. This underscores the importance of adding objective outcomes to address the limitations of subjective evaluations and provides further support for NF’s efficacy. As NF is grounded in biological principles, it is crucial to assess biologically based outcomes, extending beyond behavioral measures, to ensure a direct assessment of the underlying neural mechanisms affected by the treatment.

Indeed, studies have shown that NF can alter ERPs in both children [49] and adults with ADHD [50]. In ASD, Konicar et al. [51] demonstrated that adolescents were able to modify their brain activity through NF training. EEG recordings during training showed a reduction in Delta power and an increase in Alpha power. Moreover, these neurophysiological changes were associated with improvements in ASD symptoms, as measured by the Social Responsiveness Scale (SRS), further validating the potential of NF. However, it remains unassessed whether the applied NF- training also affected rsEEG activity as a neural signature frequently aberrant in ASD.

Changes in rsEEG activity after NF training have been assessed in various populations, such as learning-disabled children [52], adults with alcohol use disorder [53] and children with ASD [54], suggesting that NF can induce a reorganization of neural activity at rest, beyond localized adjustments at trained electrode sites. In ASD, Pineda et al. [54] found that children who underwent mu rhythm- NF training, showed decreased rs mu amplitude and coherence at central sites. Similarly, Kouijzer et al. [55] reported that children with ASD exhibited reduced rs Theta and Delta activity, along with increased resting Beta activity at central sites after having been trained with a Theta/Beta protocol. In a follow-up study, efforts to reduce Theta power in children with ASD were successful, showing decreased Theta power both during the sessions and in the post-treatment rsEEG measurements [56]. Notably, the decreases in Theta power during session data and in the rsEEG were highly correlated, suggesting that the reduction in Theta power induced by NF was maintained after treatment concluded.

Summarized, a key observation in the rsEEG literature in ASD is the consistent reduction in relative (particularly in EO paradigms [19]), as well as in absolute Alpha power [34–36] and an increased absolute Delta power [21–24]. Konicar et al. [51] demonstrated that, during training, NF can increase absolute Alpha power and decrease absolute Delta power and that these changes were associated with improvements in ASD symptomatology. Furthermore, evidence suggests that EEG changes during NF training can be sustained in the rs after treatment as shown by Kouijzer et al. [56]. Building on these results, and given our aim to compare rsEEG pre–post intervention data with previously reported changes in absolute Alpha and Delta power during SCP training (Konicar et al. [51]), we chose to retain the analysis of absolute power in the present study.

Based on these findings, we *primary* hypothesize that the NF training in Konicar et al. [51] not only increased absolute Alpha power during training, but also led to a rise in absolute rsEEG Alpha activity, particularly in the EO condition, from pre- to post- intervention in the experimental group (EG) compared to the treatment as usual (TAU) control group (CG). Further, we conduct an *exploratory investigation* regarding changes in absolute rsEEG Delta activity from pre- to post- intervention, reflecting the absolute Delta power reductions observed during training. In a similar, *exploratory manner*, to verify the validity of the findings, we explore if rsEEG brain activity correlates with the subjective, psychological level, specifically the affective state (using the Positive and Negative Affect Schedule (PANAS) [57]), as a general indicator of affective well-being and the SRS [58] as ASD-specific indicator.

## Methods and materials

### Experimental design and procedure

The present study is part of a randomized, controlled clinical intervention trial (RCT, Clinical Trial Registration: DRKS00012339) and utilizes the same sample of adolescents with ASD as previously studied in Konicar et al. [51]. The primary aim of Konicar et al. [51] was to investigate changes in ASD symptomatology, assessed through the SRS [58], following EEG-based slow cortical potential (SCP) NF training.

The SCP NF training was conducted as follows: Participants in the EG completed 24 SCP training sessions, divided into two phases of 12 sessions each, with a one-week break in between. During this break, they were encouraged to practice their individual training strategies at home. This aimed to support the transfer of learned skills to everyday life. Each participant also received a structured home training diary to record completed exercises, mental strategies, training contexts, and any behavioral changes during the break.

**Fig. 1:**
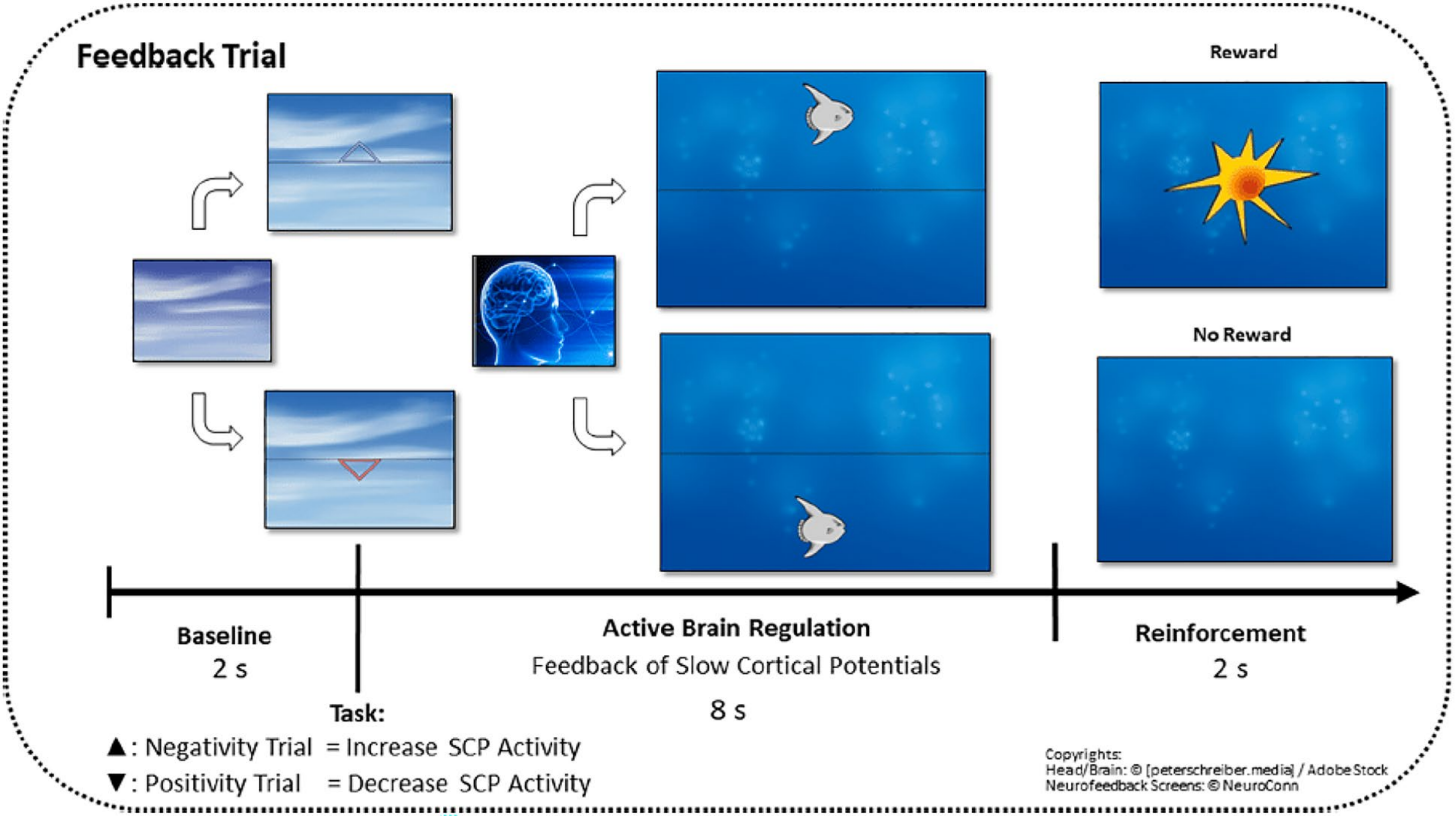
At the start of each trial, a triangle indicated the required SCP polarity: upward for negative shifts (increased cortical activation), downward for positive shifts (decreased cortical activation / inhibition). Following baseline recording, SCP activity was displayed in real time as a moving object (e.g., a fish or moon) on the participant’s screen, with upward movement reflecting increased and downward movement decreased cortical activation. Participants were instructed to volitionally control the object’s movement by producing SCP shifts in the indicated direction. Successful regulation—defined as a shift in the required polarity lasting at least 2 consecutive seconds within the final 4 s of a trial—was rewarded with a sun symbol and verbal encouragement from the trainers. Each session began with an eye movement calibration task to enable real-time artifact correction and minimize eye movement influences

For neurofeedback training, SCP activity was recorded from fronto-central brain regions (FCz) and displayed on the participants’ screen using a graphical object (see Fig. 1). Each SCP training session included 120 trials, divided into three 8-minute blocks: the first and last blocks provided feedback (SCP activity shown on the screen), while the middle block was a transfer condition without visual feedback.

NF training resulted in meaningful improvements in ASD psychopathology (e.g., reduced SRS scores) in the EG, with a slight advantage over the TAU CG (for a detailed description, see Konicar et al. [51]).

Building on the basis RCT project, the present study examines whether, alongside subjective psychological improvements, ASD-specific physiological indicators at rest also change when comparing the EG and CG. rsEEG was recorded in the week immediately before (pre, T1) and immediately after (post, T2) the interventions (NF/TAU) and serves as the primary outcome of this study.

### Participants and randomization

For the initial project, a power analysis for a repeated-measures ANOVA (*α* = 0.05, effect size = 0.25) indicated that a sample size of 40 participants would yield a power of 0.869. Given the heterogeneity of ASD and typically smaller intervention effects, an effect size of 0.25 was considered a conservative, yet clinically meaningful threshold. The resulting power was deemed sufficient to detect within-between interactions.

Therefore, a total of 41 male, right-handed adolescents (mean age: 14.05 ± 1.76 years) diagnosed with ASD were included in the current study. ASD diagnoses were confirmed through the Autism Diagnostic Interview-Revised (ADI-R, German version; [59]) and the Autism Diagnostic Observation Schedule-2 (ADOS-2, German version; [60]), which served as inclusion criteria. Exclusion criteria comprised an intelligence quotient below 70, medical conditions interfering with NF training or physiological measurements (e.g., head injuries, major Axis I psychotic disorders, severe tics, Tourette syndrome, or depression with suicidality), and prior NF experience. Pharmacological and psychosocial treatments were permitted if kept consistent throughout the study.

Included participants were randomly assigned to either the EG (*n*_*1*_ = 21), receiving SCP NF training, or to the CG (*n*_*2*_ = 20), receiving TAU, which consisted of four one-hour counseling sessions focused on well-being, development, and daily life strategies. Each session was scheduled at equal time intervals. Randomisation was performed using a structured pairwise procedure designed to minimise bias. Specifically, every two individuals who enrolled consecutively were paired (sequential pairing). For each pair, the names of the two participants were written on separate slips of paper, sealed in identical envelopes, and placed into a box. A researcher or designated staff member then blindly selected one envelope. According to the predefined allocation rule, the participant whose name was drawn first was assigned to the EG, while the other was assigned to the CG (sequence generation). This randomization process was repeated for each subsequent pair until the target group sizes were reached, ensuring unbiased allocation and preventing any subjective influence on group assignment.

The study was approved by the Ethics Committee of the Medical University of Vienna and conducted in line with the Declaration of Helsinki. Written informed consent was obtained from both participants and their legal guardians during the enrolment phase. Data collection was a part of a randomized controlled clinical study involving the same population (Clinical Trial Registration: DRKS00012339).

### Resting state EEG recordings: objective, psychophysiological measures

Brain activity at rest before (pre, T1) and after (post, T2) interventions was recorded via Ag/AgCl electrodes using ABRALYT HiCl paste from 19 active electrode sites (Fp1, FP2, F3, F4, F7, F8, Fz, FCz, C3, C4, Cz, P3, P4, Pz, T5, T6, O1, O2, and Oz) according to the 10–20 system. The recordings were made using the Theraprax EEG System (NeuroConn, Ilmenau, Germany). The vertical electrooculogram (VEOG) was recorded using two electrodes placed at the outer canthi of the eyes, with the left and right mastoids serving as ground and reference electrodes. All recordings were conducted with a 128 Hz sampling rate and electrode impedances were constantly kept below 5 kΩ. For the eight-minute rsEEG, participants were asked to relax during two different recording conditions: (a) 4 min with open eyes (O) and (b) 4 min with closed eyes (C). These conditions alternated every minute in the following order: (C)→(O)→(C)→(O), followed by the reverse order: (O)→(C)→(O)→(C). During the EO condition, participants were asked to look at a fixation cross in in the center of the screen, while during the EC condition, the participants were similarly instructed to direct their gaze straight ahead as if focusing on the center of the screen.

### Clinical psychopathology and affective well-being: subjective, psychological measures

For the *exploratory* investigation of the link between the objective, physiological and the subjective psychological level, the data from the pre and post measurements of the SRS scale [58], a well-validated parental report for assessing ASD- specific psychopathology, was used. In addition to the total score, which serves as an index of overall deficiency in social communication and interaction, five subscales can be differentiated: Social Awareness (SA), Social Cognition (SCOG), Social Communication (SCOM), Social Motivation (SMOT), and Restricted Interests and Repetitive Behavior (Autistic Mannerism; AM). Items are rated on a 4-point Likert scale ranging from 0 (“not true”) to 3 (“almost always true”), with a score of 3 indicating the highest level of symptom severity. Higher scores on the total and subscale levels indicate greater impairment in the corresponding domains.

The German version of the PANAS [57] was used to assess affective well-being, based on pre- and post-measurements. This self-report includes 20 adjectives reflecting positive and negative affect dimensions. Participants rate the extent to which each adjective applies to them on a scale from 1 (“not at all”) to 5 (“extremely”), with higher scores indicating stronger expressions of the respective affect. Positive affect correlates with social activity and satisfaction, while negative affect is linked to stress and health issues [61].

### Preprocessing and data analysis

All rsEEG data were processed and analyzed using Brain Vision Analyzer 2.2 (BVA) [62]. Following bandpass filtering (1–70 Hz) and a 50 Hz notch filter, the raw data were semi-automatically inspected and corrected for artifacts based on predefined criteria: the maximum allowed voltage step was set to 50 µV within a ± 200 ms window around events. A minimum activity threshold of 0.5 µV was applied to defined intervals and the maximum allowed difference in values within intervals was limited to 200 µV over a 200 ms interval. Ocular correction was performed using Independent Component Analysis (ICA) based on the Infomax restricted algorithm in BVA. In addition, ocular artifacts were corrected offline following the method by Gratton et al. [63]. Subsequently, a Fourier Transformation (10% segment length; Hanning window) was applied to the segmented data. Finally, segmented data was averaged for each condition (pre, post; EO, EC) for each participant. Frequency bands of interest, namely Alpha (8–12.9 Hz) and Delta (0.5–3.9 Hz), were extracted and calculated as absolute power for frontocentral regions (Fp1, FP2, F3, F4, F7, F8, Fz, FCz, C3, C4, Cz). This resulted in a total of 30 EEG data sets (16 rsEEG data sets from the EG and 14 rsEEG data sets from the CG) for primary statistical analysis.

### Statistical analysis

Statistical analyses were conducted using SPSS 27 [64]. For the *primary analysis*, namely the absolute frontocentral Alpha in the EO condition, a repeated-measures analysis of variance (ANOVA) was applied, with the within-subject factor *Time* (pre = T1 / post = T2 measures) and the between-subject factor *Group* (EG/CG). For the *exploratory analysis on the physiological level*, namely the frontocentral absolute Delta power in the EO and EC condition, a repeated-measures ANOVA was similarly applied with the within-subject factor *Time* (pre = T1 / post = T2 measures) and the between-subject factor *Group* (EG / CG). *T*-tests were conducted to investigate differences between the groups at baseline and to assess changes from pre to post intervention within the EG and CG separately. To examine potential relationships between the psychophysiological level (T1: baseline Alpha activity, EO) and the subjective-psychological level (T1: SRS total and subscale scores) in an *explorative manner*, Pearson’s correlations were calculated. In a similar, *exploratory vein*, further possible relationships between the psychophysiological level (T1: baseline Alpha/Delta activity, EO/EC) and the subjective-psychological level (T1: PANAS item score) were investigated using Pearson’s correlations. Possible relationships between changes on the two levels (changes in EEG and questionnaire data, i.e., post (T2) minus pre (T1) scores) were also explored using Pearson’s correlations in the same *exploratory* manner. For both: the primary analysis (absolute Alpha power in the EO condition) and all exploratory analyses, *p*-values ≤ 0.05 were considered statistically significant. While uncorrected *p*-values are reported for exploratory analyses in the main text, Benjamini-Hochberg corrected *p*-values [65] are additionally provided in the Supplementary Material.

### Large language models

ChatGPT [66] was utilized in the creation of this manuscript to refine the linguistic expression.

## Results

### Resting state EEG

#### Alpha band activity

Repeated-measures ANOVA revealed a significant *Time* (pre, post) x *Group* (EG, CG) effect (*F*(1,32) = 6,086; *p* =.019; *η²*= 0.160) regarding changes in Alpha band power in the EO condition. Post-hoc tests revealed an increase in Alpha power in the EG from before (*m* = 8, 38, *SD* = 3,45) to after intervention (*m* = 9,75, *SD* = 4,91), but did not reach the level of significance (*t*_(16)=_ -1.882, *p* =.078). In the CG, a decrease in Alpha power was observable from before (*m* = 13, 28, *SD* = 5,75) to after intervention (*m* = 11,99 *SD* = 5,39) (*t*_(16)=_ 1,635, *p* =.122). A trend for a difference in Alpha band power at baseline before interventions was found (*t*_(35)=_ 1,891, *p* =.067).

**Fig. 2:**
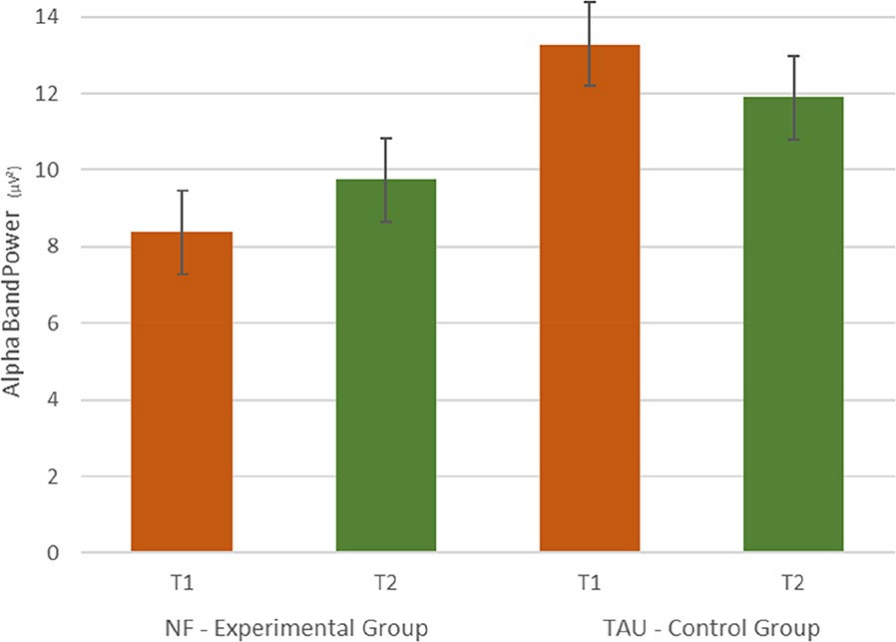
Increases in Alpha band from before (T1, red bars) to after intervention (T2, green bars) in the NF - EG; Decreases in Alpha band from before (T1, red bars) to after intervention (T2, green bars) in the TAU - CG

#### Delta band activity

**Fig. 3:**
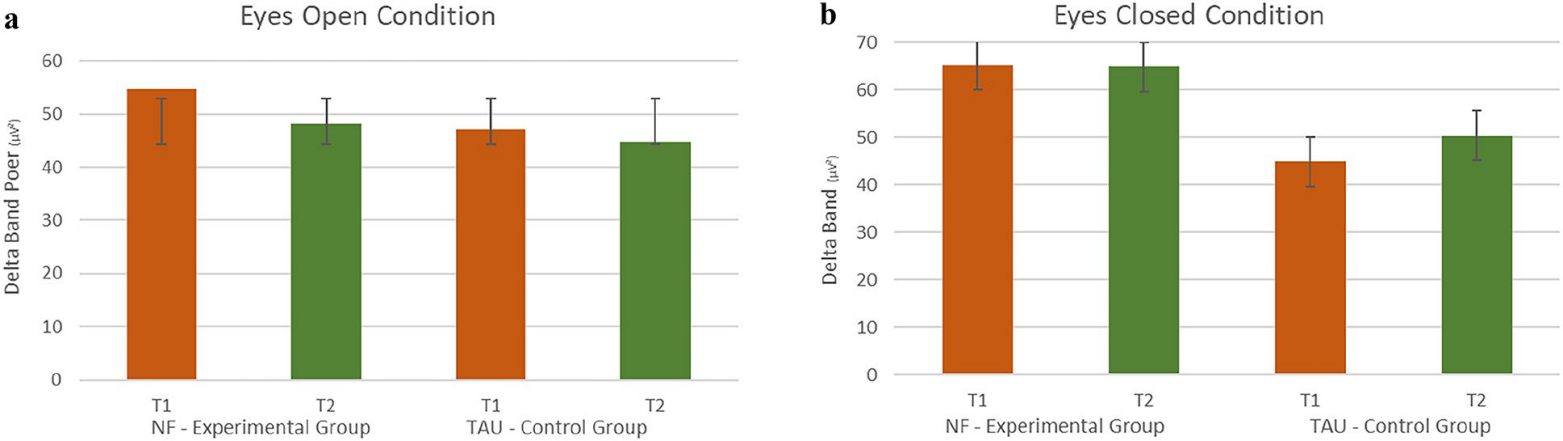
Changes in Delta band from before (T1, red bars) to after intervention (T2, green bars) in the NF - EG and the TAU– CG for the EO Condition on the left side (**a**) and for the EC Condition on the right side (**b**)

Exploratory repeated-measures ANOVA investigating possible changes in Delta frequency band yielded a significant effect of *Time* (*F*(1,28) = 4,87; *p* =.036; *η²*= 0.148) for the EO condition (but not for the EC condition), indicating that EO - Delta Power decreased in both groups from before (EG: *m* = 54,69, *SD* = 13,39; CG: *m* = 47,17, *SD* = 17,98) to after (EG: *m* = 48,23, *SD* = 12,84; CG: *m* = 44,75, *SD* = 15,47) intervention (see Fig. 3a).

A trend for a *Time* (pre, post) x *Group* (EG, CG) interaction in the EC condition (*F*(1,28) = 3,50; *p* =.072; *η²*= 0.111) was found. Post-hoc tests revealed a decrease in Delta power in the EG, which reached significance only in the EO condition (EO: *t*_(16)_ = 2.27, *p* =.037; EC: *t*_(17)_ = 2.93, *p* =.959) from before (EO: *m* = 54,69, *SD* = 13,38; EC: *m* = 65, 24, *SD* = 22,35) to after intervention (EO: *m* = 48,23, *SD* = 12,84; EC: *m* = 64,84, *SD* = 31,44). In the CG, a non-significant slight decrease in Delta power was observed in the EO condition (EO: t(16) = 1.177, *p* =.257) from before (EO: m = 47.17, SD = 17.96) to after intervention (EO: m = 44.75, SD = 15.47), as shown in Fig. 3a. In contrast, in the EC condition, a non-significant increase in Delta power was observed (EC: t(13) = -1.377, *p* =.192), with Delta power increasing from before (EC: m = 44.81, SD = 11.31) to after intervention (EC: m = 50.31, SD = 16.99), as shown in Fig. 3b.

### Relationships between RsEEG and autism spectrum disorder psychopathology (SRS-2)

**Fig. 4:**
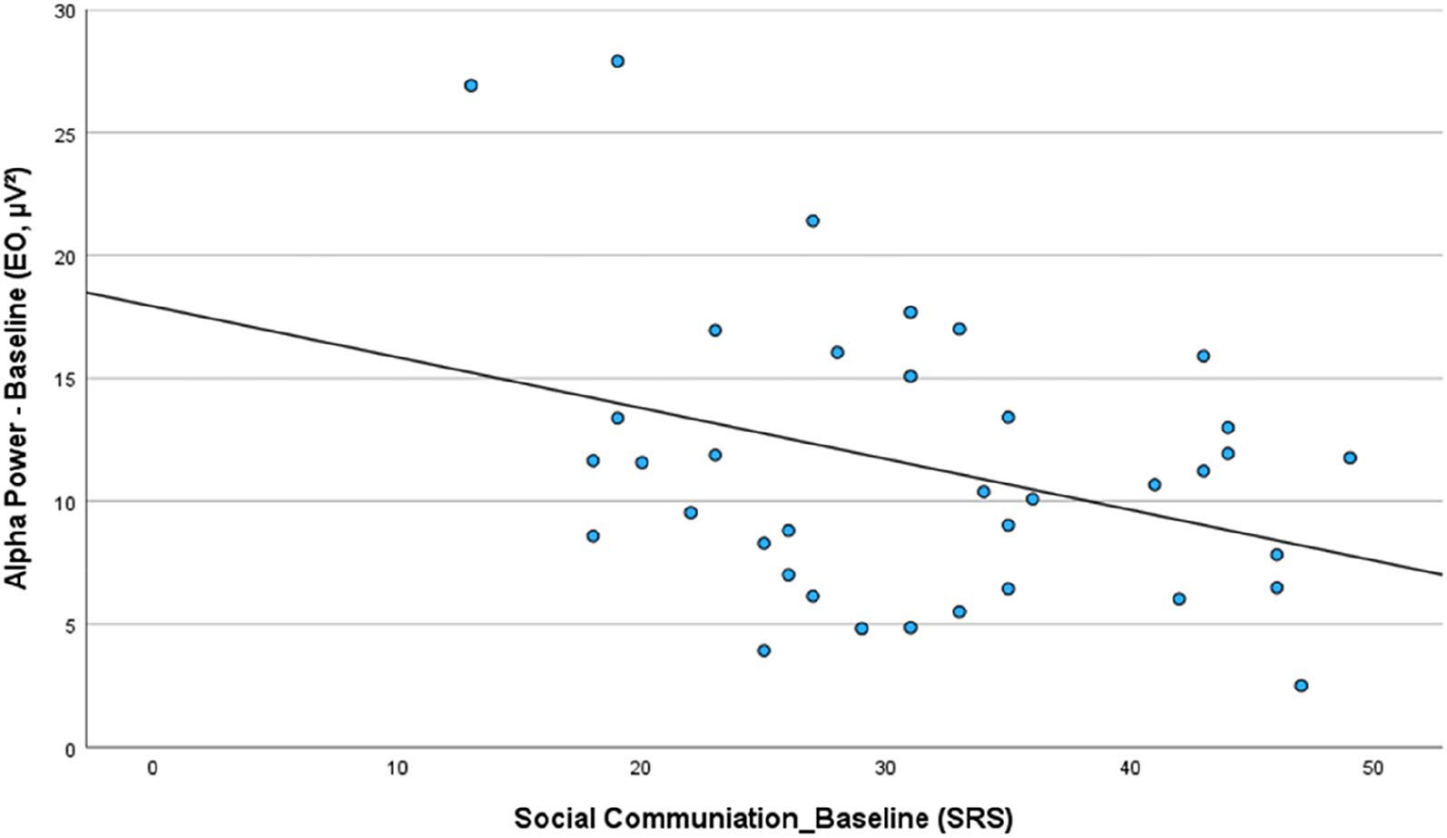
Correlation between *SRS* - *Social Communication* subscale at baseline and EO - rs Alpha Power (µV²) at baseline. The higher the SRS score (ASD psychopathology) at baseline, the lower the Alpha power at baseline

Exploratory analyses investigating the relationships between baseline ASD symptomatology (SRS scores) and rs EO Alpha activity at baseline suggest considerable links between the subjective-psychological and physiological levels. The trend for a relationship between the *SRS Total score* (*r* = −.303, *p* =.069), is strengthened by two more trends, one on the *SRS - Autistic Mannerism* subscale (*r* = −.303, *p* =.068) and the other on the *SRS - Social Cognition* subscale (*r* = −.301, *p* =.070), but more prominently by a significant relationship with the *SRS* - *Social Communication* subscale (*r* = −.347, *p* =.036). This pattern indicates that more severe forms of ASD are related to less Alpha Power at baseline, as depicted in Fig. 4.

Additional analyses regarding possible relationships between rs Delta power at baseline and SRS scores yielded no significant results. Further exploratory analyses investigating possible relationships between changes in rsEEG data (Alpha, Delta) and changes in SRS scores did not yield significant results. For results regarding changes in SRS scores from before to after SCP - NF training see Konicar et al. [51].

### Relationships between RsEEG and positive and negative emotions (PANAS)

**Fig. 5:**
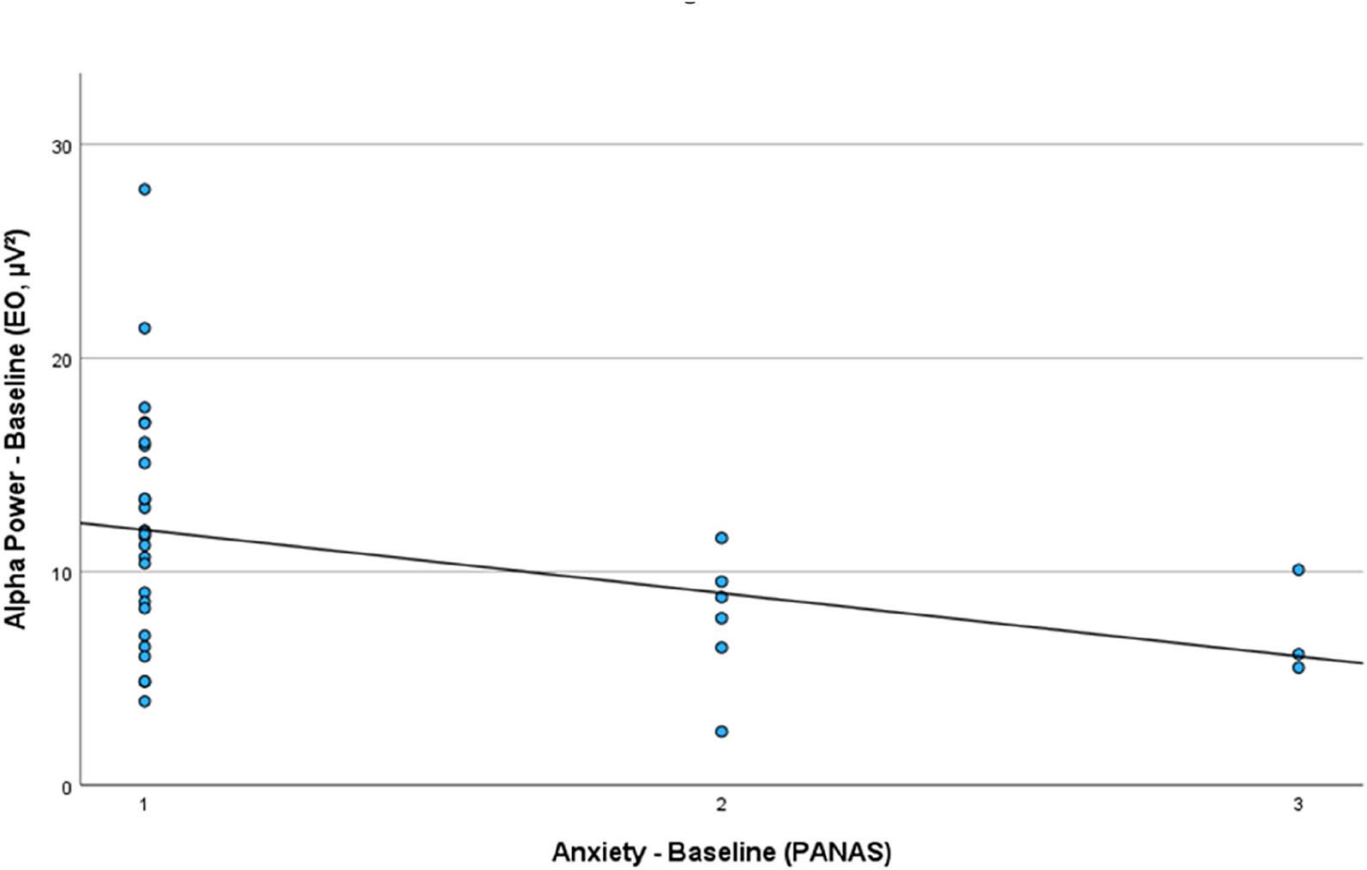
Correlation between Anxiety Baseline (PANAS) and EO - Baseline Alpha Power (µV²). The higher the *PANAS* - *Anxiety* score at baseline, the lower the Alpha power at baseline

Exploratory analyses regarding the relationships between baseline global, positive and negative affect (PANAS) and rsEEG activity at baseline results in the following picture: Baseline Alpha Power was found to be linked to baseline *PANAS - Anxiety* (EO: *r* = −.357, *p* =.033, EC: *r* = −.330, *p* =.060), as depicted in Fig. 5 and baseline *PANAS - Ashamed* (EC: *r* =.350, *p* =.049).

Regarding Delta activity at baseline, relationships were found with baseline *PANAS - Enthusiastic* (EC: *r* = −.320, *p* =.069) and baseline *PANAS– Upset* (EC: *r* =.321, *p* =.064).

This pattern indicates that the lower Alpha power is at baseline, the higher the self-reported scores in anxiety and the lower the self-reported scores in shame. On the other hand, higher Delta Power at baseline is linked to higher annoyance and less enthusiasm in general. No significant results were found for Total scores of Positive and Negative Affect.

**Fig. 6:**
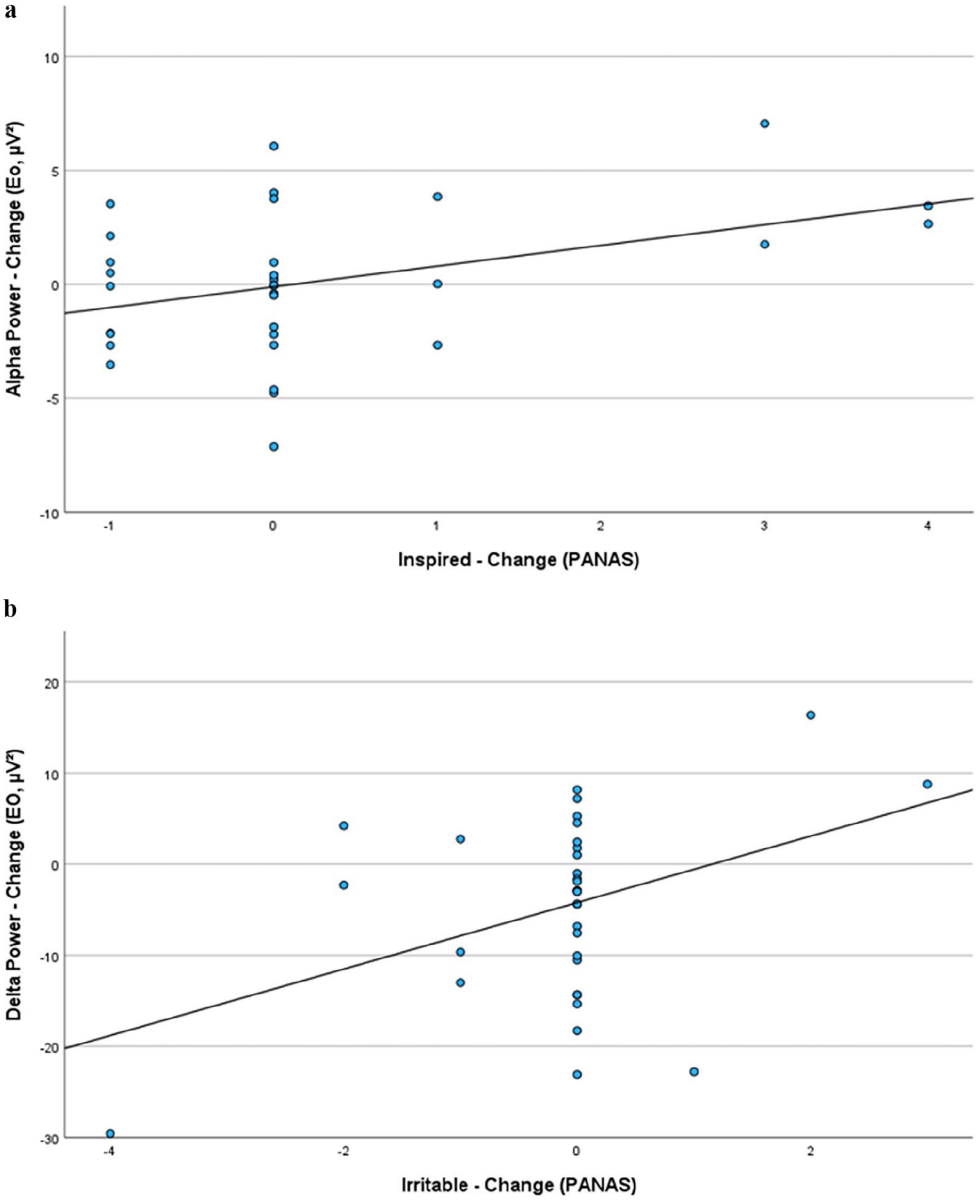
Correlation between Changes in Alpha and Delta power (post minus pre) and changes in PANAS– affects (post minus pre). **a** The more Alpha power is increasing from before to after intervention, the more the positive affect *Inspired* is also increasing. **b** The more Delta power is decreasing from before to after intervention, the more the negative affect *Irritable* is decreasing

Additionally, exploratory analyses investigating the relationships between changes in positive and negative affects and changes in rsEEG activity (Alpha, Delta) point towards the following: the more Alpha power is increasing from before to after interventions, the more also the *PANAS affects Inspired* (EO: *r* =.394, *p* =.026, as depicted in Fig. 6a), *Active* (EC: *r* =.427, *p* =.015) and *Interested* (EC: *r* =.339, *p* =.057) increased, while *Excited* decreased (EO: *r* = −.354, *p* =.043) from before to after interventions, independent of the specific intervention.

Regarding changes from Delta power, exploratory analyses point towards a different picture: The more Delta power decreased from before to after interventions, the more also the *PANAS - affects Hostile* (EO: *r* =.386, *p* =.026), *Irritable* (EO: *r* =.399, *p* =.021) and *Nervous* (EO: *r* =.315, *p* =.079) decreased, while the *PANAS - affects Strong* (EC: *r* = −.394, *p* =.026) and *Enthusiastic* (EC: *r* = −.358, *p* =.044) increased from before to after interventions, independent of the specific intervention.

## Discussion

In the present study, we investigated rsEEG changes before and after SCP NF-training in adolescents with ASD, comparing the EG receiving NF with a TAU CG. We hypothesized that the EEG changes observed during the NF training sessions in the main study [51]—specifically, the increase in Alpha power - would be sustained in the rsEEG measurements of the EG, indicating enduring alterations in brain activity patterns. Additionally, we explored whether rsEEG activity was correlated with subjective self-report measures, specifically the PANAS and the SRS in an explorative manner.

We found that, in the EO condition, absolute Alpha power changes from pre- to post-intervention differed significantly between the EG and CG. While Alpha power in the CG decreased slightly, the EG expectedly exhibited an increase, though marginally not significant. Delta power decreased from pre- to post-intervention in both groups, with the reduction reaching statistical significance only in the EG, confirming our hypothesis.

Interestingly, the modulation of rs oscillatory activity through SCP NF has also been examined in a further study by Konicar et al. [67], which reported an increase in relative alpha and a decrease in relative delta power following training. Moreover, the participants—offenders with psychopathy—exhibited reduced self-reported aggression and approach behavior, along with increased behavioral inhibition and error sensitivity post-training. These findings suggest that oscillatory modulation via NF may enhance inhibitory control and contribute to the stabilization of cortical networks.

Alpha oscillations, in particular, have been closely linked to inhibitory neural processes [68]. In ASD, the frequently reported reduction in Alpha power may reflect disruptions in these inhibitory mechanisms. Additionally, increased low-frequency activity—such as elevated Delta power—may represent a compensatory mechanism aimed at counteracting excessive high-frequency excitatory activity driven by inhibitory dysfunction [37]. This lack of inhibition may result in a brain state that is more susceptible to sensory overload, less able to flexibly allocate attention, and less capable of adapting to changing environmental demands—all prominent features of ASD. By enhancing volitional control over SCPs, NF may directly modulate these oscillatory imbalances, supporting more flexible and balanced cortical functioning.

Adding to this, SCP training has been shown to increase contingent negative variation [49], an ERP component reflecting enhanced anticipatory attention and cognitive preparation, both associated with inhibitory control mechanisms. Moreover, SCP training has been found to be correlated with increased activation in the anterior cingulate cortex [69], a region central to attentional regulation and top-down inhibitory processes. Together, these neurophysiological changes may foster more adaptive top-down regulation by strengthening inhibitory networks, contributing to the normalization of EEG patterns—such as increased alpha activity and a reduction in compensatory delta power—indicative of more stable and regulated cortical activity.

Taken together, these findings position SCP training as a promising biological intervention for conditions like ASD, where disrupted inhibitory control and atypical oscillatory activity play a central role in the disorder’s neurophysiological profile.

Furthermore, our findings support the conclusion by Neo et al. [19], which highlights the utility of specifically EO paradigms in utilizing rsEEG as a biomarker for ASD. Additionally, our results suggest that four minutes of EO recordings may be sufficient to detect significant effects. In addition, the observed changes in rsEEG activity align with the findings of Kouijzer et al. [56], who showed that NF-induced EEG changes during training could persist in rs measures after treatment. This suggests that NF may induce lasting alterations in brain activity, warranting further research to explore its long-term effects on symptomatology. However, the marginal significance of our Alpha power findings highlights the need for additional research to confirm these effects and explore the mechanisms underlying the maintenance of training-induced EEG changes in rs. Importantly, our study expands the existing literature, which has assessed significant changes solely in directly trained frequencies in ASD [52–54], by demonstrating that SCP NF may also induce broader, lasting changes in neural activity that extend beyond the targeted frequency bands. Future studies will be needed to explore the underlying mechanisms responsible for these effects.

Finally, the self-report measures offered valuable insights into the relationship between rsEEG activity and both ASD-specific symptoms and affective states. SRS scores demonstrated that greater severity of ASD symptoms was linked to lower Alpha power at baseline in the EO condition. Furthermore, analysis of PANAS scores revealed that both, lower baseline Alpha power and higher baseline Delta power correlated with increased negative affect. These findings contribute important information to previous research that has linked ASD to reduced Alpha and increased Delta power at rest, by suggesting that more severe symptoms may be associated with more pronounced alterations in these frequency bands. Interestingly, this seems to work both ways, as changes in rsEEG activity from pre- to post-intervention were associated with affective improvements: increases in Alpha power were linked with enhanced positive affect, whereas reductions in Delta power corresponded with decreases in negative affect. The observed relationship between rsEEG changes and affective improvements further supports the notion that reduced Alpha and increased Delta power at rest are associated with ASD symptom severity and that NF may help shift brain activity towards more typical patterns, thereby alleviating ASD symptomatology.

Nevertheless, one primary challenge in clinical NF research is still the experimental design, particularly in selecting suitable control and sham feedback conditions [70]. We chose to include an active CG because it offers both practical and ethical advantages. To still control for non-specific effects as thoroughly as possible, we administered the Fragebogen zur Erfassung relevanter Therapiebedingungen (FERT; [71]), a self-report measure designed to assess relevant treatment conditions, such as participant expectations and participant - therapist interactions (see [51]; questionnaire results are available in the Supplementary Materials), which yielded stable levels of these non-specific factors across participants. However, potential confounding factors remain, including varying treatment environments, which could not be fully controlled within the study design. Additionally, while baseline differences in rs Alpha power were not statistically significant, the EG exhibited lower Alpha power. Given the assessed association between reduced Alpha power and higher SRS scores, this suggests that the EG may have included participants with more severe ASD symptoms, potentially influencing the observed effects. Future studies should therefore aim to address these challenges by implementing more complex experimental designs and larger sample sizes, which could mitigate confounding factors as well as baseline differences and yield statistically more robust results. Another limitation is the gender bias in our sample, as the study focused exclusively on male adolescents with ASD. This limits the applicability of the findings to females with ASD, who may demonstrate distinct patterns of brain activity. To enhance the generalizability of the results, future research should aim to include female participants as well.

To the best of our knowledge, this is the first study to correlate self- and parental reports (PANAS, SRS) with rsEEG data pre- and post-NF intervention in ASD. Additionally, the significant correlations between rsEEG changes and improvements in self-reported symptoms lend further validation to an intervention like NF, which is susceptible to non-specific effects [46]. These findings provide robust support for EO rsEEG as a reliable biological outcome measure in ASD research, which additionally offers a naturalistic, less intrusive assessment of neural functioning compared to task-related EEG and minimizes the variability that arises from individual differences in task perception across the spectrum. Furthermore, our study highlights the importance of integrating both subjective and objective outcome measures to enhance their respective validity. It also underscores the need to consider not only parent or teacher-rated reports of symptomatology, but especially self-report measures, as the ultimate goal is to improve the subjective quality of life of individuals affected with ASD.

## Conclusions

In conclusion, our findings suggest that SCP NF may induce lasting changes in rsEEG activity in adolescents with ASD, particularly in Alpha and Delta power, which align with improvements in affective states and ASD symptomatology. The correlation between rsEEG changes and self-reported measures further supports the potential of NF as an effective intervention for ASD. However, challenges remain regarding experimental design, gender bias, and baseline differences, highlighting the need for future research with larger, more diverse samples and refined methodologies. Overall, this study emphasizes the value of integrating both, objective rsEEG measures and subjective self-reports to comprehensively assess NF interventions in ASD.

## Electronic supplementary material

Below is the link to the electronic supplementary material.

Supplementary Material 1

## Data Availability

The datasets used and/or analysed during the current study are available from the corresponding author on reasonable request.

## Abbreviations

ADHD: Attention deficit hyperactivity disorder
ADI-R: Autism diagnostic interview-revised
ADOS-2: Autism Diagnostic Observation Schedule-2
ANOVA: Analysis of Variance
CG: Control group
EC: Eyes closed
EG: Experimental group
EO: Eyes open
ERP: Event-related potentials
MRI: Magnetic resonance imaging
NF: Neurofeedback
PANAS: Positive and Negative Affect Scale
RS: Resting state
SCP: Slow cortical potential
SRS: Social Responsiveness Scale
TAU: Treatment as usual
