# Supplementary material for: Multi-level treatment outcome evaluation in adolescents with autism spectrum disorder
Source: Child Adolesc Psychiatry Ment Health. 2025 May 19;19:58. doi: 10.1186/s13034-025-00909-1 (PMC12090636; doi:10.1186/s13034-025-00909-1)
Supplement: Supplementary file 1 — Supplementary Material 1 [file 13034_2025_909_MOESM1_ESM.docx]

**SUPPLEMENTARY MATERIAL**

**Multi-level treatment outcome evaluation**

**in adolescents with Autism Spectrum Disorder**

Resting state EEG and subjective-psychological indicators

before and after a randomised, controlled neurofeedback training trial

Auer, Gabriel Anton^1^, Plener, Paul Lukas^1,2.3,4^, Poustka, Luise^5^ & Konicar, Lilian^1,2,3*^

1 Department of Child and Adolescent Psychiatry, Medical University of Vienna, Vienna, Austria

2 Comprehensive Center for Pediatrics (CCP), Medical University of Vienna, Vienna, Austria

3 Comprehensive Center for Clinical Neuroscience and Mental Health (C3NMH), Medical

University of Vienna, Vienna, Austria

4 Department of Child and Adolescent Psychiatry and Psychotherapy, Ulm University, Ulm, Germany

5 Department of Child and Adolescent Psychiatry, University Hospital Heidelberg, Heidelberg, Germany

**1. Additional Analyses and Corrections**

**Ad. 4.2a) Additional Analysis and corrections regarding Relationships between absolute rs Alpha Power (EO) and SRS-2**

| **Clin-Psy Questionnaires / Subscale** | **N** | **Pearson Correlation** | **Effect size** (Cohen, 1992) | **Significance**  ***p*-values uncorrected**  (2-sided) | ***p*-values**  **BH-adjusted**  (2-sided) |
| --- | --- | --- | --- | --- | --- |
| Social Communication (SCOM) | 37 | -0,347 | *medium* | 0,036* | 0,1050 |
| Restricted Interests and Repetitive Behavior (Autistic Mannerism; AM) | 37 | -0,303 | *small-medium* | 0,068^T^ | 0,1050 |
| SRS-2 Total | 37 | -0,303 | *small-medium* | 0,069^T^ | 0,1050 |
| Social Cognition (SCOG) | 37 | -0,301 | *small-medium* | 0,070^T^ | 0,1050 |
| Social Awareness (SA) | 37 | -0,161 | *small* | 0,342 | 0,4104 |
| Social Motivation (SMOT) | 37 | 0,066 | *small* | 0,698 | 0,6980 |

**Ad. 4.2b). Additional Analysis and corrections regarding relationships between absolute rs Delta Power Power and SRS-2**

As no significant results were obtained regarding the potential relationships between absolute rs Delta Power Power (EO+EC) and SRS-2, as well as regarding changes in rsEEG data (Alpha, Delta) and changes in SRS-2 scores, no further analysis or corrections were conducted.

**Ad. 4.3.1a) Relationships between absolute rs Alpha EO at baseline and PANAS at baseline**

| **Clin-Psy Questionnaires / Subscale** | **N** | **Pearson Correlation** | **Effect size** (Cohen, 1992) | **Significance**  ***p*-values uncorrected**  (2-sided) | ***p*-values**  **BH-adjusted**  (2-sided) |
| --- | --- | --- | --- | --- | --- |
| PANAS_BASELINE_anxious | 36 | -,357^*^ | *medium* | 0,033* | 0,720 |
| PANAS_BASELINE_confused | 36 | 0,184 | *small* | 0,283 | 0,995 |
| PANAS_BASELINE_concerned | 36 | -0,183 | *small* | 0,286 | 0,995 |
| PANAS_BASELINE_enthusiastic | 35 | 0,177 | *small* | 0,309 | 0,995 |
| PANAS_BASELINE_active | 36 | -0,147 | *small* | 0,393 | 0,995 |
| PANAS_BASELINE_ashamed | 35 | 0,105 | *small* | 0,548 | 0,995 |
| PANAS_BASELINE_attentive | 35 | 0,094 | - | 0,590 | 0,995 |
| PANAS_BASELINE_awake | 35 | -0,081 | - | 0,646 | 0,995 |
| PANAS_BASELINE_excited | 34 | -0,080 | - | 0,652 | 0,995 |
| PANAS_BASELINE_joyful | 36 | 0,078 | - | 0,652 | 0,995 |
| PANAS_BASELINE_strong | 36 | -0,065 | - | 0,708 | 0,995 |
| PANAS_BASELINE_proud | 34 | -0,061 | - | 0,733 | 0,995 |
| PANAS_BASELINE_irritated | 35 | 0,059 | - | 0,738 | 0,995 |
| PANAS_BASELINE_annoyed | 36 | -0,040 | - | 0,817 | 0,995 |
| PANAS_BASELINE_hostile | 36 | 0,039 | - | 0,823 | 0,995 |
| PANAS_BASELINE_frightened | 36 | 0,038 | - | 0,824 | 0,995 |
| PANAS_BASELINE_interested | 36 | 0,027 | - | 0,877 | 0,995 |
| PANAS_BASELINE_guilty | 36 | 0,022 | - | 0,896 | 0,995 |
| PANAS_BASELINE_nervous | 35 | 0,016 |  | 0,930 | 0,995 |
| PANAS_BASELINE_Positive Affect | 36 | -0,013 | - | 0,939 | 0,995 |
| PANAS_BASELINE_determined | 35 | -0,005 | - | 0,976 | 0,995 |
| PANAS_BASELINE_Negative Affect | 36 | 0,001 | - | 0,995 | 0,995 |

**Ad. 4.3.1.b) Relationships between absolute rs Alpha EC at baseline and PANAS at baseline**

| **Clin-Psy Questionnaires / Subscale** | **N** | **Pearson Correlation** | **Effect size** (Cohen, 1992) | **Significance**  ***p*-values uncorrected**  (2-sided) | ***p*-values**  **BH-adjusted**  (2-sided) |
| --- | --- | --- | --- | --- | --- |
| PANAS_BASELINE_ashamed | 32 | ,350^*^ | *medium* | 0,049* | 0,665 |
| PANAS_BASELINE_anxious | 33 | -0,33 | *medium* | 0,060^T^ | 0,665 |
| PANAS_BASELINE_awake | 32 | -0,30 | *small* | 0,096 | 0,703 |
| PANAS_BASELINE_enthusiastic | 33 | 0,267 | *small* | 0,134 | 0,734 |
| PANAS_BASELINE_strong | 33 | -0,195 | *small* | 0,278 | 0,953 |
| PANAS_BASELINE_proud | 31 | -0,159 | *small* | 0,394 | 0,953 |
| PANAS_BASELINE_nervous | 32 | -0,138 | *small* | 0,453 | 0,953 |
| PANAS_BASELINE_frightened | 33 | 0,132 | *small* | 0,465 | 0,953 |
| PANAS_BASELINE_interested | 33 | 0,124 | *small* | 0,493 | 0,953 |
| PANAS_BASELINE_hostile | 33 | 0,122 | *small* | 0,499 | 0,953 |
| PANAS_BASELINE_attentive | 32 | 0,115 | *small* | 0,532 | 0,953 |
| PANAS_BASELINE_confused | 33 | 0,087 | - | 0,628 | 0,953 |
| PANAS_BASELINE_annoyed | 33 | -0,083 | - | 0,645 | 0,953 |
| PANAS_BASELINE_concerned | 33 | -0,076 | - | 0,672 | 0,953 |
| PANAS_BASELINE_determined | 32 | 0,057 | - | 0,756 | 0,953 |
| PANAS_BASELINE_excited | 32 | -0,044 | - | 0,812 | 0,953 |
| PANAS_BASELINE_guilty | 33 | -0,035 | - | 0,845 | 0,953 |
| PANAS_BASELINE_irritated | 33 | -0,028 | - | 0,879 | 0,953 |
| PANAS_BASELINE_Positive Affect | 33 | -0,022 | - | 0,903 | 0,953 |
| PANAS_BASELINE_Negative Affect | 33 | -0,016 | - | 0,931 | 0,953 |
| PANAS_BASELINE_joyful | 33 | 0,012 | - | 0,948 | 0,953 |
| PANAS_BASELINE_active | 33 | -0,011 | - | 0,953 | 0,953 |

**Ad. 4.3.2a) Relationships between absolute rs Delta EC at baseline and PANAS at baseline**

| **Clin-Psy Questionnaires / Subscale** | **N** | **Pearson Correlation** | **Effect size**  (Cohen, 1992) | **Significance**  ***p*-values uncorrected**  (2-sided) | ***p*-values**  **BH-adjusted**  (2-sided) |
| --- | --- | --- | --- | --- | --- |
| PANAS_BASELINE_annoyed | 34 | 0,321 | *medium* | 0,064^T^ | 0,761 |
| PANAS_BASELINE_enthusiastic | 33 | -0,320 | *medium* | 0,069^T^ | 0,761 |
| PANAS_BASELINE_strong | 34 | -0,262 | *small* | 0,134 | 0,962 |
| PANAS_BASELINE_guilty | 34 | -0,228 | *small* | 0,194 | 0,962 |
| PANAS_BASELINE_proud | 32 | -0,223 | *small* | 0,221 | 0,962 |
| PANAS_BASELINE_Positive Affect | 34 | -0,192 | *small* | 0,277 | 0,962 |
| PANAS_BASELINE_determined | 33 | -0,163 | *small* | 0,363 | 0,962 |
| PANAS_BASELINE_hostile | 34 | 0,120 | *small* | 0,499 | 0,962 |
| PANAS_BASELINE_confused | 34 | -0,112 | *small* | 0,529 | 0,962 |
| PANAS_BASELINE_attentive | 33 | -0,110 | *small* | 0,543 | 0,962 |
| PANAS_BASELINE_anxious | 34 | 0,098 | - | 0,581 | 0,962 |
| PANAS_BASELINE_nervous | 33 | -0,090 | - | 0,620 | 0,962 |
| PANAS_BASELINE_joyful | 34 | -0,061 | - | 0,730 | 0,962 |
| PANAS_BASELINE_interested | 34 | -0,057 | - | 0,750 | 0,962 |
| PANAS_BASELINE_irritated | 33 | -0,052 | - | 0,772 | 0,962 |
| PANAS_BASELINE_active | 34 | -0,050 | - | 0,780 | 0,962 |
| PANAS_BASELINE_ashamed | 33 | -0,046 | - | 0,800 | 0,962 |
| PANAS_BASELINE_excited | 33 | -0,037 | - | 0,839 | 0,962 |
| PANAS_BASELINE_Negative Affect | 34 | 0,032 | - | 0,856 | 0,962 |
| PANAS_BASELINE_awake | 33 | -0,029 | - | 0,874 | 0,962 |
| PANAS_BASELINE_frightened | 34 | -0,009 | - | 0,961 | 0,989 |
| PANAS_BASELINE_concerned | 34 | -0,002 | - | 0,989 | 0,989 |

**Ad. 4.3.2b) Relationships between absolute rs Delta EO at baseline and PANAS at baseline**

As no significant results were obtained regarding the potential relationships between absolute rs Delta EO at baseline and PANAS at baseline, no further analysis or corrections were conducted.

**Ad. 4.3.3a) Relationships between changes in absolute rs Alpha EO (post-pre) and changes in PANAS (post - pre)**

| **Clin-Psy Questionnaires / Subscale** | **N** | **Pearson Correlation** | **Effect size**  (Cohen, 1992) | **Significance**  ***p*-values uncorrected**  (2-sided) | ***p*-values**  **BH-adjusted**  (2-sided) |
| --- | --- | --- | --- | --- | --- |
| PANAS_CHANGE_excited | 32 | ,394* | *medium* | 0,026* | 0,475 |
| PANAS_CHANGE_joyful | 33 | -,354* | *medium* | 0,043* | 0,475 |
| PANAS_CHANGE_frightened | 33 | 0,304 | *small* | 0,086 | 0,629 |
| PANAS_CHANGE_interested | 33 | 0,236 | *small* | 0,185 | 0,662 |
| PANAS_CHANGE_confused | 33 | 0,235 | *small* | 0,188 | 0,662 |
| PANAS_CHANGE_hostile | 33 | 0,231 | *small* | 0,197 | 0,662 |
| PANAS_CHANGE_guilty | 33 | 0,224 | *small* | 0,211 | 0,662 |
| PANAS_CHANGE_determined | 32 | -0,207 | *small* | 0,255 | 0,701 |
| PANAS_CHANGE_ashamed | 32 | 0,158 | *small* | 0,387 | 0,952 |
| PANAS_CHANGE_awake | 31 | 0,146 | *small* | 0,433 | 0,952 |
| PANAS_CHANGE_Negative Affect | 33 | 0,137 | *small* | 0,446 | 0,952 |
| PANAS_CHANGE_strong | 33 | 0,123 | *small* | 0,495 | 0,952 |
| PANAS_CHANGE_enthusiastic | 33 | -0,118 | *small* | 0,513 | 0,952 |
| PANAS_CHANGE_annoyed | 33 | -0,079 | - | 0,661 | 0,952 |
| PANAS_CHANGE_irritated | 33 | 0,079 | - | 0,663 | 0,952 |
| PANAS_CHANGE_concerned | 32 | -0,070 | - | 0,703 | 0,952 |
| PANAS_CHANGE_active | 33 | 0,061 | - | 0,735 | 0,952 |
| PANAS_CHANGE_nervous | 32 | 0,042 | - | 0,819 | 0,986 |
| PANAS_CHANGE_Positive Affect | 33 | 0,034 | - | 0,851 | 0,986 |
| PANAS_CHANGE_proud | 32 | 0,013 | - | 0,943 | 0,989 |
| PANAS_CHANGE_anxious | 33 | 0,005 | - | 0,977 | 0,989 |
| PANAS_CHANGE_attentive | 32 | -0,002 | - | 0,989 | 0,989 |

**Ad. 4.3.3b) Relationships between changes in absolute rs Alpha EC (post-pre) and changes in PANAS (post - pre)**

| **Clin-Psy Questionnaires / Subscale** | **N** | **Pearson Correlation** | **Effect size**  (Cohen, 1992) | **Significance**  ***p*-values uncorrected**  (2-sided) | ***p*-values**  **BH-adjusted**  (2-sided) |
| --- | --- | --- | --- | --- | --- |
| PANAS_CHANGE_active | 32 | 0,427* | *medium* | 0,015* | 0,327 |
| PANAS_CHANGE_interested | 32 | 0,339 | *medium* | 0,057^T^ | 0,631 |
| PANAS_CHANGE_frightened | 32 | -0,209 | *small* | 0,251 | 0,858 |
| PANAS_CHANGE_nervous | 31 | -0,211 | *small* | 0,255 | 0,858 |
| PANAS_CHANGE_awake | 30 | -0,214 | *small* | 0,256 | 0,858 |
| PANAS_CHANGE_attentive | 30 | 0,213 | *small* | 0,257 | 0,858 |
| PANAS_CHANGE_guilty | 32 | -0,184 | *small* | 0,314 | 0,858 |
| PANAS_CHANGE_proud | 30 | -0,177 | *small* | 0,350 | 0,858 |
| PANAS_CHANGE_Positive Affect | 32 | 0,161 | *small* | 0,379 | 0,858 |
| PANAS_CHANGE_concerned | 31 | -0,145 | *small* | 0,436 | 0,858 |
| PANAS_CHANGE_ashamed | 31 | 0,140 | *small* | 0,453 | 0,858 |
| PANAS_CHANGE_annoyed | 32 | -0,129 | *small* | 0,482 | 0,858 |
| PANAS_CHANGE_Negative Affect | 32 | -0,122 | *small* | 0,507 | 0,858 |
| PANAS_CHANGE_joyful | 32 | 0,094 | - | 0,610 | 0,917 |
| PANAS_CHANGE_excited | 32 | 0,062 | - | 0,736 | 0,917 |
| PANAS_CHANGE_irritated | 32 | 0,058 | - | 0,751 | 0,917 |
| PANAS_CHANGE_strong | 32 | 0,057 | - | 0,757 | 0,917 |
| PANAS_CHANGE_confused | 32 | 0,050 | - | 0,784 | 0,917 |
| PANAS_CHANGE_anxious | 32 | -0,048 | - | 0,792 | 0,917 |
| PANAS_CHANGE_enthusiastic | 32 | 0,038 | - | 0,838 | 0,921 |
| PANAS_CHANGE_determined | 31 | -0,020 | - | 0,914 | 0,958 |
| PANAS_CHANGE_hostile | 32 | -0,006 | - | 0,973 | 0,973 |

**Ad. 4.3.3a) Relationships between changes in absolute rs Delta EO (post-pre) and changes in PANAS (post - pre)**

| **Clin-Psy Questionnaires / Subscale** | **N** | **Pearson Correlation** | **Effect size**  (Cohen, 1992) | **Significance**  ***p*-values uncorrected**  (2-sided) | ***p*-values**  **BH-adjusted**  (2-sided) |
| --- | --- | --- | --- | --- | --- |
| PANAS_CHANGE_irritated | 33 | ,399* | *medium* | 0,021* | 0,291 |
| PANAS_CHANGE_hostile | 33 | ,386* | *medium* | 0,026* | 0,291 |
| PANAS_CHANGE_nervous | 32 | 0,315 | *medium* | 0,079^T^ | 0,330 |
| PANAS_CHANGE_annoyed | 33 | 0,303 | *small* | 0,087 | 0,330 |
| PANAS_CHANGE_enthusiastic | 33 | 0,303 | *small* | 0,087 | 0,330 |
| PANAS_CHANGE_Negative Affect | 33 | 0,300 | *small* | 0,090 | 0,330 |
| PANAS_CHANGE_determined | 32 | 0,269 | *small* | 0,137 | 0,430 |
| PANAS_CHANGE_ashamed | 32 | 0,258 | *small* | 0,154 | 0,424 |
| PANAS_CHANGE_joyful | 33 | -0,232 | *small* | 0,194 | 0,447 |
| PANAS_CHANGE_frightened | 33 | 0,227 | *small* | 0,203 | 0,447 |
| PANAS_CHANGE_active | 33 | -0,156 | *small* | 0,385 | 0,728 |
| PANAS_CHANGE_anxious | 33 | 0,152 | *small* | 0,397 | 0,728 |
| PANAS_CHANGE_strong | 33 | 0,141 | *small* | 0,435 | 0,736 |
| PANAS_CHANGE_excited | 32 | 0,124 | *small* | 0,500 | 0,786 |
| PANAS_CHANGE_concerned | 32 | -0,086 | - | 0,641 | 0,882 |
| PANAS_CHANGE_attentive | 32 | -0,079 | - | 0,667 | 0,882 |
| PANAS_CHANGE_proud | 32 | -0,075 | - | 0,681 | 0,882 |
| PANAS_CHANGE_interested | 33 | -0,073 | - | 0,685 | 0,837 |
| PANAS_CHANGE_Positive Affect | 33 | 0,048 | - | 0,792 | 0,917 |
| PANAS_CHANGE_guilty | 33 | 0,030 | - | 0,869 | 0,955 |
| PANAS_CHANGE_confused | 33 | -0,019 | - | 0,917 | 0,955 |
| PANAS_CHANGE_awake | 31 | -0,010 | - | 0,955 | 0,955 |

| **Clin-Psy Questionnaires / Subscale** | **N** | **Pearson Correlation** | **Effect size**  (Cohen, 1992) | **Significance**  ***p*-values uncorrected**  (2-sided) | ***p*-values**  **BH-adjusted**  (2-sided) |
| --- | --- | --- | --- | --- | --- |
| PANAS_CHANGE_strong | 32 | -,394* | *medium* | 0,026* | 0,487 |
| PANAS_CHANGE_enthusiastic | 32 | -,358* | *medium* | 0,044* | 0,487 |
| PANAS_CHANGE_active | 32 | -0,285 | *small* | 0,114 | 0,774 |
| PANAS_CHANGE_Positive Affect | 32 | -0,268 | *small* | 0,137 | 0,774 |
| PANAS_CHANGE_nervous | 31 | 0,249 | *small* | 0,176 | 0,774 |
| PANAS_CHANGE_hostile | 32 | 0,214 | *small* | 0,240 | 0,832 |
| PANAS_CHANGE_concerned | 31 | -0,198 | *small* | 0,286 | 0,832 |
| PANAS_CHANGE_joyful | 32 | -0,175 | *small* | 0,339 | 0,832 |
| PANAS_CHANGE_proud | 30 | -0,180 | *small* | 0,340 | 0,832 |
| PANAS_CHANGE_confused | 32 | 0,137 | *small* | 0,455 | 0,962 |
| PANAS_CHANGE_anxious | 32 | 0,105 | *small* | 0,567 | 0,962 |
| PANAS_CHANGE_attentive | 30 | 0,100 | *small* | 0,598 | 0,962 |
| PANAS_CHANGE_guilty | 32 | -0,095 | - | 0,606 | 0,962 |
| PANAS_CHANGE_excited | 32 | 0,073 | - | 0,690 | 0,962 |
| PANAS_CHANGE_irritated | 32 | -0,063 | - | 0,734 | 0,962 |
| PANAS_CHANGE_awake | 30 | -0,060 | - | 0,752 | 0,962 |
| PANAS_CHANGE_frightened | 32 | -0,046 | - | 0,801 | 0,962 |
| PANAS_CHANGE_annoyed | 32 | -0,034 | - | 0,855 | 0,962 |
| PANAS_CHANGE_determined | 31 | 0,033 | - | 0,862 | 0,962 |
| PANAS_CHANGE_Negative Affect | 32 | 0,027 | - | 0,882 | 0,962 |
| PANAS_CHANGE_interested | 32 | -0,016 | - | 0,930 | 0,962 |
| PANAS_CHANGE_ashamed | 31 | 0,009 | - | 0,962 | 0,962 |

**Ad. 4.3.3a) Relationships between changes in absolute rs Delta EC (post-pre) and changes in PANAS (post - pre)**

**REFERENCES**

**Cohen, J.** (1992). Statistical Power Analysis. *Current Directions in Psychological Science*, *1*(3), 98-101. https://doi.org/10.1111/1467-8721.ep10768783.
